# A Review of Friction Performance of Lubricants with Nano Additives

**DOI:** 10.3390/ma14216310

**Published:** 2021-10-22

**Authors:** Muhammad Waqas, Rehan Zahid, Muhammad Usman Bhutta, Zulfiqar Ahmad Khan, Adil Saeed

**Affiliations:** 1 Campus H-12, School of Mechanical & Manufacturing Engineering (SMME), National University of Sciences & Technology (NUST), Islamabad 44000, Pakistan; waqas130562@outlook.com (M.W.); rehanzahid@smme.nust.edu.pk (R.Z.); usmanbhutta@smme.nust.edu.pk (M.U.B.); 2 NanoCorr, Energy & Modelling (NCEM) Research Group, Department of Design & Engineering, Talbot Campus, Bournemouth University, Fern Barrow, Poole BH12 5BB, UK; asaeed4@bournemouth.ac.uk

**Keywords:** nanoparticles, friction, wear, tribology, biolubricants, synthetic oils, lubrication mechanisms, agglomeration

## Abstract

It has been established in literature that the addition of nanoparticles to lubricants at an optimum concentration results in a lower coefficient of friction compared to lubricants with no nanoparticle additives. This review paper shows a comparison of different lubricants based on the COF (coefficient of friction) with nanoadditives. The effect of the addition of nanoparticles on the friction coefficient was analyzed for both synthetic and biolubricants separately. The limitations associated with the use of nanoparticles are explained. The mechanisms responsible for a reduction in friction when nanoparticles are used as an additive are also discussed. Various nanoparticles that have been most widely used in recent years showed good performance within lubricants, including CuO (copper oxide), MoS_2_ (molybdenum disulfide), and TiO_2_ (titanium dioxide). The paper also indicates some research gaps that need to be addressed.

## 1. Introduction

The high friction and wear in engine components results in major dissipation of engine power. Almost 17–19% of an engine’s generated power is used to overcome friction [1]. In recent years, due to an increase in the number of vehicles on the road, the fuel demand has been increased [2]. In order to overcome this rise in fuel demand, the automobile industry is continuously working to produce vehicles with better fuel economy by improving the tribological performance of engines. In an internal combustion engine, lubricant oil plays a vital role in achieving better fuel savings through a reduction in the friction and wear of interacting surfaces. Over the past few decades, several studies have been conducted to improve lubrication systems, so that the friction and wear in engine components can be reduced. With the recent advancements of nanotechnology, nanomaterials have emerged as a potential source to enhance the tribo-performance of different lubrication systems [3]. With this, a number of experimental investigations have been performed to evaluate the effects of the addition of nanoparticles to different lubricants. Due to their extremely small size, nanoparticles fill the valleys on the interacting surfaces, resulting in a reduction in friction and wear [4]. It has also been found that nanoparticles tend to develop a three-body rolling influence between the interacting surfaces, which results in friction reduction [5]. Experiments have shown that when nanoparticles are used in optimum concentrations, they also improve the anti-wear (AW) and extreme pressure (EP) characteristics of lubricants [6]. Moreover, nanoparticles have better thermal conductivity compared to the base oil, which improves the stability of tribopairs by taking away the heat generated by friction. The nanoparticles mostly used are environmentally friendly as they tend to minimize the use of the certain additives that have a hazardous impact on the environment [7].

To date, a number of nanoparticles have been used with various base oils, and the effects on the wear and friction performance of these base oils have been studied [8,9,10,11]. Those nanomaterials that are most used as lubricant additives include graphene [12,13,14], graphene oxide [15], carbon nanotubes [16,17], boron nitride [18], silicon dioxide [19], copper oxide [20], and titanium dioxide [21]. Additionally, a few studies have also been conducted on combinations of different nanomaterials (hybrid nanoparticles) as lubricant additives [22,23,24]. In these studies, nanoparticles in different concentrations were used under varying operating conditions. The nanoparticle concentrations and operating conditions were varied to identify the optimum concentrations of the nanoparticles for a specific lubricant under specific operating conditions. At optimum concentration, the friction and wear were found to be minimal compared to base oils without nanoparticles.

**Figure 1:**
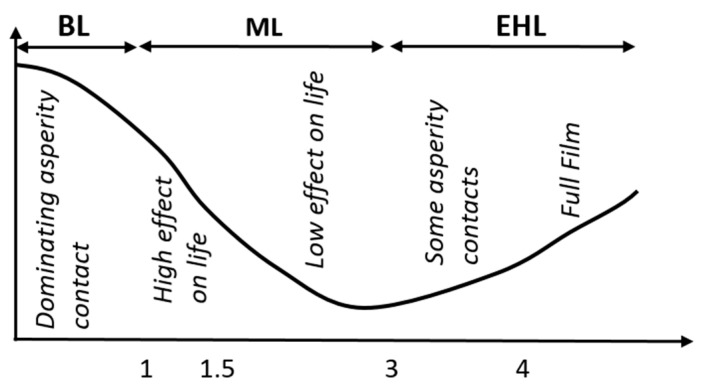
Classification of the lubrication regime based on the lambda ratio. BL, body lubrication; ML, mixed lubrication; EL, elastohydrodynamic lubrication.

It has already been established in the literature that the lubrication regime between two interacting surfaces can be categorized into mixed and boundary lubrication and elastohydrodynamic/hydrodynamic lubrication regimes, depending on the film thickness ratio, also known as the lambda (λ) ratio, as shown in Figure 1 [25], where the λ-ratio is defined as the ratio between the minimum film thickness and the composite surface roughness. High friction and wear in the boundary lubrication regime due to asperities contact result in a major loss of energy, which makes the use of additives essential this regime [26]. In order to decrease friction and wear for various lubrication conditions, there is a need to modify the already available lubricants. In this regard, the use of nanolubricants in internal combustion engines has been the focus of many researchers, leading to improvements in the tribological characteristics of the interacting surfaces [27].

Although a large number of experimental studies have been conducted in order to evaluate the effect of the addition of nanoparticles on the tribological characteristics, there are still certain areas that are yet to be explored in order to fully understand the effects of nanoparticles on the performance of lubricant oils. This review discusses the nanomaterials used as additives in the last five years and compares their effect on the friction performance of different synthetic and biolubricants in detail. Only those nanoparticles with sufficient data available are considered. This article also explains the mechanisms responsible for the anti-wear and antifriction behavior of nanolubricants, along with the issues involved in the stability of nanoparticles in lubricants.

## 2. Lubrication Mechanisms of Nanoparticles

**Figure 2:**
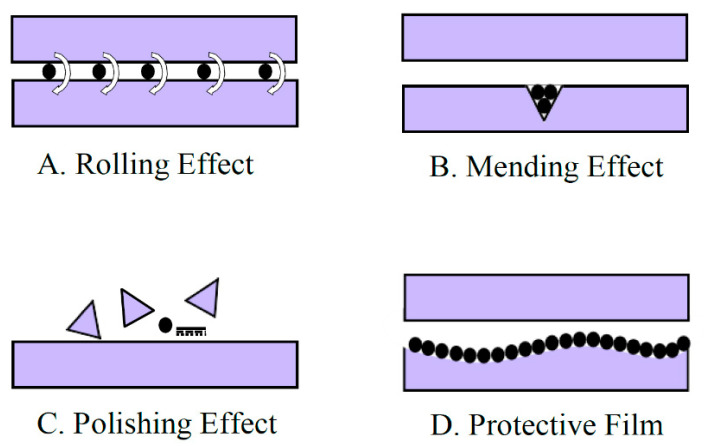
Lubrication mechanisms of nanoparticles. (**A**) Rolling Effect; (**B**) Mending Effect; (**C**) Polishing Effect; (**D**) Protective Film.

In the literature, several mechanisms responsible for the reduction in friction and wear have been discussed. These mechanisms vary for different nanoparticles [28]. It has been found that the adsorption of nanocomposite carbon additives on the interacting surface enhances the tribological performance of SN/GF-5 lubricants [17]. WS_2_ (tungsten disulfide) nanoparticles form a protective layer and cause a patching effect, making the interacting surfaces smoother, which in turn leads to improved tribological properties of lubricant oil [29]. An experimental study showed the formation of chemical bonds between MWCNTs (multi-walled carbon nanotubes) and nanoparticles of CuO when used with 10w-40 oil. TiO_2_ nanoparticles have the potential to produce both primary and the secondary effects when they are used in lubricant oil as additives. Primary effects refer to the formation of a protective film between the interacting surfaces where nanoparticles produce a ball-bearing effect, thus changing sliding friction into rolling friction. Meanwhile, secondary effect refers to the compensation of the wear loss produced due to rubbing of the interacting surfaces by deposition of nanoparticles on wear scars [30]. The mechanisms responsible for the improved tribological performance when nanoparticles are used as additives are categorized as: (1) Effect of rolling, (2) effect of mending, (3) effect of polishing, and (4) effect of the tribofilm (protective film) formation [31,32]. In rolling or ball-bearing effects, nanoparticles produce a three-body rolling effect between the interacting surfaces, converting sliding friction into rolling friction, as shown in Figure 2A. Mending effects refer to the deposition of nanoparticles, filling the valleys and grooves on the interacting surface, as displayed in Figure 2B. Polishing effects refer to the breaking of large asperities on the surface and making it smooth, as shown in Figure 2C. In the tribofilm formation mechanism, nanoparticles form a protective film between the interacting surfaces, which helps to prevent direct contact of these surfaces, as shown in Figure 2D. Quasi-spherical and spherical nanoparticles generally exhibit a rolling effect between the interacting surfaces. When a nanolubricant containing such nanoparticles is employed between the interacting bodies, positive results can be obtained owing to the rolling of such nanoparticles between the interacting surfaces. Mending effect also known as self-sustaining effect, in which nanoparticles adhere to the interacting surfaces and tend to fill the valleys on the interacting surfaces, thus resulting in lowering in surface roughness. Polishing effect is also known as smoothing effect, which is mostly produced by the nanolubricants containing hard nanoparticles. Such nanoparticles tend to break large asperities, which accumulate in the valleys of the interacting surfaces, thus resulting in a smooth surface. In tribofilm formation, a chemical reaction between the interacting surfaces and the medium between the interacting surfaces take place, and, as a result, a chemical layer is formed on the interacting surfaces [3].

## 3. Nanoparticles in Synthetic Lubricants

In the last five years, a number of nanomaterials have been used as additives in synthetic lubricants. The effect of the addition of these nanomaterials on the friction performance of synthetic lubricants is discussed in this section. This study is focused only on those nanomaterials that have been used by different researchers in their experimentations.

### 3.1. TiO_2_ Nanoparticles

**Figure 3:**
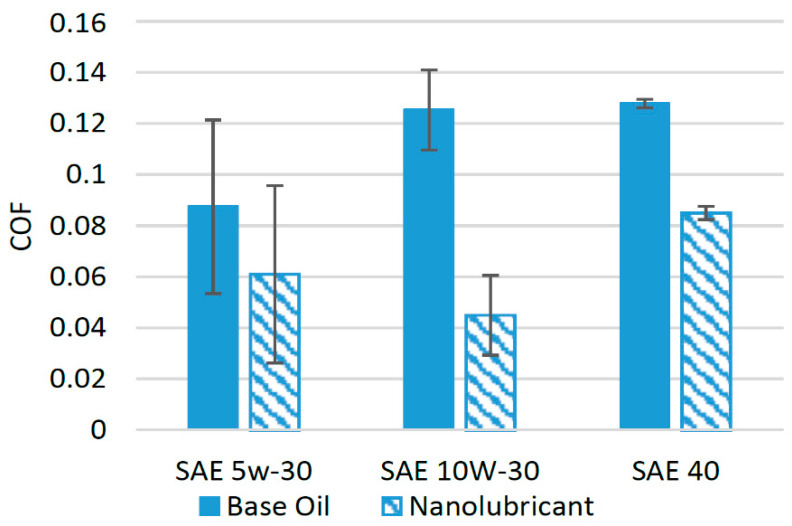
Effect of TiO_2_ NPs on the friction performance of synthetic lubricants.

Various studies in recent years have been devoted to the use of TiO_2_ nanoparticles in different synthetic oils [33,34,35,36]. The effect of the addition of nanoparticles on the friction performance of several lubricants is shown in Figure 3. TiO_2_ nanoparticles were added in different concentrations in SAE 5w-30 oil, and tribological tests with pin-on-disk configuration were conducted under different operating conditions. The results of the experiments showed that the coefficient of friction (COF) decreased by almost 30% compared to base oils when optimum concentration of the nanoparticles was used [21]. In another study, when TiO_2_ nanoparticles were used as an additive in SAE 5w-30 at an optimum concentration of 0.25%, tested with a pin-on-ring configuration, the COF was reduced by 48% compared to base oil [35]. Meanwhile, the COF was reduced by almost 35% when TiO_2_ nanoparticles were used in SAE 10w-30 at an optimum concentration of 0.5% [37]. The addition of nanoparticles in a 0.01% volume concentration improved the COF of SAE 40 engine oil by almost 1.5% compared to pure SAE 40 in a four-ball tribotester [38]. This positive behavior of TiO_2_ nanoparticles can be attributed to the conversion of sliding friction to rolling friction due to the presence of nearly spherical nanoparticles at the interface of the interacting bodies [39]. It can be seen from Figure 3 that among the other lubricants, TiO_2_ performed exceptionally well with SAE 10w-30 and reduced the COF by a greater percentage, which shows that TiO_2_ nanoparticles are much more compatible with SAE 10w-30 oil compared to other oils.

### 3.2. Graphene (Gr) Nanoparticles

**Figure 4:**
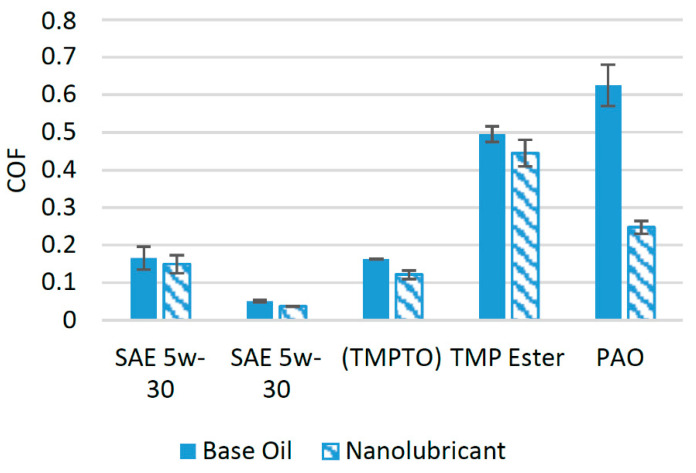
Effect of Gr NPs on the friction performance of synthetic lubricants.

Graphene nanoparticles and graphene nanosheets have been considered as a friction modifier by many researchers [40]. In a previous study, graphene nanoparticles were used as an additive in SAE 5w-30 at different concentrations and were tested using a ball-on-plate tribotester. It was found that the optimum concentration of graphene nanoparticles for SAE 5w-30 is 0.10 wt%, and at this concentration, the COF was reduced by almost 10%. The reduction in the COF when using Gr nanoparticles was attributed to the tribofilm formation between the interacting surfaces. Gr nanosheets also enhanced the performance of 5w-30 lubricant oil and reduced the COF by almost 28%. This reduction in the COF was attributed to the ability of Gr nanoparticles to fill the valleys between asperities [4]. Figure 4 illustrates a comparison of the COF, when Gr nanoparticles and Gr nanosheets were used as additives in different lubricants. Trimethylolpropane trioleate (TMPTO) was used as a lubricant in a study where Gr nanoparticles were used as an additive. It was reported that when 0.25 wt% of Gr nanoparticles was employed as an additive, the friction performance of the base oil was enhanced by almost 25% compared to base oil when the sample was tested on a tribotester with a ball-on-plate configuration [41]. This positive effect on the tribological properties of the lubricant was because of the accumulation of nanoparticles in the valleys between the asperities of the interacting surfaces. The COF was reduced by almost 10% when 0.10 wt% of Gr nanoparticles was used as an additive in trimethylolpropane ester (TMP ester). Gr nanoparticles increased the oxidative stability of the base oil, resulting in a thicker and the stronger tribofilm between the interacting surfaces [42]. Polyalphaolefin (PAO) was used as a base lubricant in a previous study to find the effects of Gr nanoparticles on its tribological properties, and it was found that when only 0.01 wt% of Gr nanoparticles was used as an additive, the COF was reduced by almost 142% [14]. The positive interaction between the base oil and Gr nanoparticles resulted in the formation of an effective tribofilm. Moreover, the Gr nanoparticles filled the valleys between asperities and were adsorbed on the interacting surfaces, resulting in a lower coefficient of friction [14]. A comparison of the effect of the addition of graphene nanoparticles on the COF related to different lubricant oils showed that graphene nanoparticles are more suitable to be used with PAO, because the reduction in the COF of the interacting surfaces was much higher compared to that of other nanolubricants.

### 3.3. Copper (Cu) Nanoparticles

**Figure 5:**
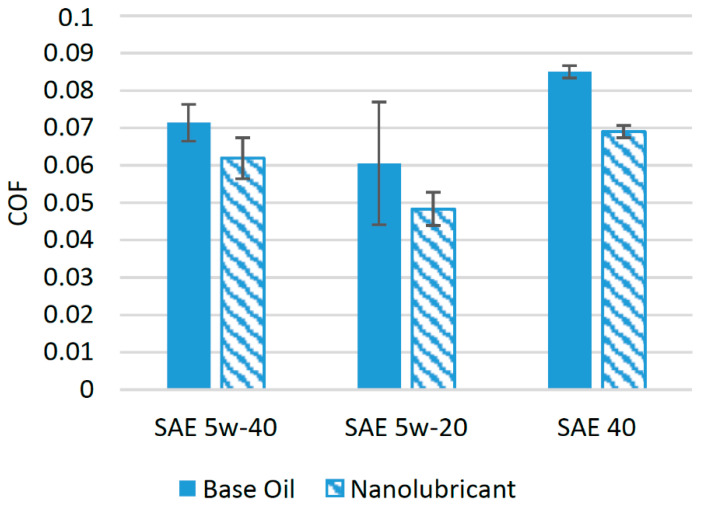
Effect of Cu NPs on the friction performance of synthetic lubricants.

A few studies have reported the use of copper nanoparticles with synthetic lubricants [43,44]. Figure 5 shows the effect of the addition of nanoparticles on the friction performance of different synthetic lubricants. It has been reported that when copper nanoparticles are employed as an additive in SAE 5w-40 oil at an optimum concentration of 0.8 wt%, the resulting COF is reduced by almost 13% when tests are conducted on a four-ball tribotester. In same study, Cu nanoparticles were also used as additives in SAE 5w-20 oil, and it was found that when 1.6 wt% of Cu nanoparticles were added, the COF was reduced by 28.5% [45]. It has also been reported that the COF of the lubricant oil SAE 40 is improved by a factor of 14% when Cu nanoparticles at an optimum concentration of 0.01 vol% are used in a four-ball tribotester [38].

The improvement in the tribological characteristics of lubricant oil by adding Cu nanoparticles can be attributed to the spherical morphology of Cu nanoparticles, which transforms sliding friction into rolling friction, producing a ball-bearing effect between interacting surfaces [46]. Among the lubricants in which Cu nanoparticles have been used, a positive effect on the COF is much prominent for SAE 5w-20 compared to other lubricants, which shows that Cu nanoparticles are more compatible with this lubricant oil compared to others.

### 3.4. Zinc Oxide (ZnO) Nanoparticles

**Figure 6:**
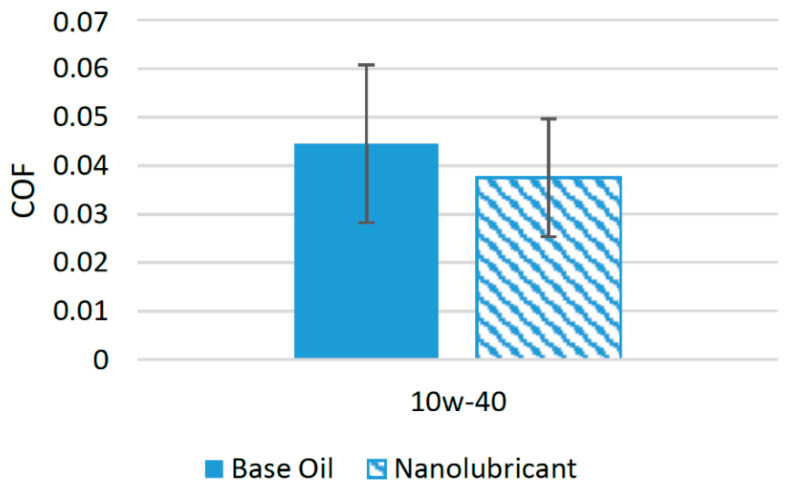
Effect of ZnO NPs on the friction performance of synthetic lubricants.

Zinc oxide nanoparticles have been used as a potential source to reduce the friction of lubricants in recent years [47]. Not much research has been conducted to test the suitability of ZnO nanoparticles with different lubricants. In a previous study, ZnO nanoparticles in different concentrations were used as additives in SAE 10w-40. The concentration of the nanoparticles ranged between 0.1 and 0.8 wt%. The results of the tribological tests with a pin-on-disk configuration showed that 0.6 wt% gave the optimum results and reduced the friction of the interacting surfaces by 22% compared to lubricant oil with no nanoparticle additives [48]. Figure 6 displays the effect of the addition of nanoparticles on the friction performance. ZnO nanoparticles have ability to transform sliding friction to rolling friction, which is the major reason for their positive effect on the tribological properties. Moreover, ZnO nanoparticles also helped in development of lubricating layers on the interacting surfaces, which resulted in a reduced COF compared to oil with no nanoparticle additives [49]. Only a few researchers have used ZnO nanoparticles in their studies to check their suitability as a friction modifier. Thus, there is a need to check the suitability of these nanoparticles with different synthetic lubricants in order to evaluate their effect on the COF and to find a lubricant oil with which these nanoparticles show their best performance.

### 3.5. Hexa-Boron Nitride (h-BN) Nanoparticles

A significant number of experimental studies have been performed to evaluate the effects of h-BN nanoparticles on different lubricants [50]. These studies have concluded that h-BN nanoparticles can be used as friction and wear modifiers, as they influence the tribological properties of different lubricants in a positive manner. In an experimental study, h-BN nanoparticles were used with SAE 5w-30 at a concentration of 0.5 wt%. The results of the tribological test using a four-ball tribotester showed that the COF was reduced by 10% at this concentration [51]. In another study, h-BN nanoparticles were used as an additive to evaluate their effects on the friction performance of polyalphaolefin (PAO). Tribological experiments with a pin-on-disk configuration were conducted at an optimum weight concentration of 0.5 wt% of h-BN nanoparticles. The results showed that when h-BN nanoparticles were used at this optimum concentration, the COF of the rubbing surfaces was reduced by almost 5.4% compared to PAO with no nanoparticle additive [52]. The effects of h-BN nanoparticles on the tribological performance of SAE 20w-50 were investigated by a group of researchers. In this experimental research, h- BN nanoparticles were used at three different concentrations 1 wt%, 2 wt%, and 3 wt% in a four-ball tribotester. The minimum value of the COF was achieved when 3 wt% of h-BN nanoparticles was employed as an additive. At this optimum concentration, the COF was reduced by almost 26.8% compared to oil with no nanoparticle additive [8]. The friction performance of SAE 15w-40 was greatly enhanced by the addition of h-BN nanoparticles. In an experimental study, 0.5 vol% of h-BN nanoparticles was used as an additive. The results of tribological tests with a four-ball configuration showed that when h-BN nanoparticles were used at this optimum concentration, the COF was reduced by almost 50% [18]. This synergetic behavior of h-BN nanoparticles shows that they can be used as friction modifiers owing to their positive effect on the friction performance of different lubricants.

**Figure 7:**
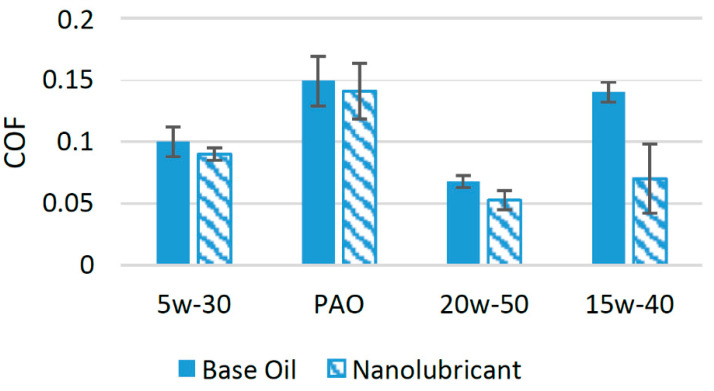
Effect of h-BN NPs on the friction performance of synthetic lubricants.

This positive effect of the h-BN nanoparticles on the friction performance is due to the formation of a boron-oxide tribofilm on the interacting surface because of tribochemical reactions [18]. Moreover, the high thermal conductivity of h-BN nanoparticles helps to carry away the heat generated due to rubbing, helping the lubricant to maintain its viscosity, which ultimately results in less metal-to-metal contact and a reduction in friction and wear [53]. Figure 7 shows a comparison of the COF of different lubricants with and without the addition of h-BN nanoparticles. Figure 7 shows that the h-BN nanoparticles performed extremely well compared to the other lubricants and decreased the COF in the case of SAE 15w-40 by a huge percentage, which shows that these nanoparticles are highly compatible with SAE 15w-40.

### 3.6. Molybdenum Disulfide (MoS_2_) Nanoparticles

**Figure 8:**
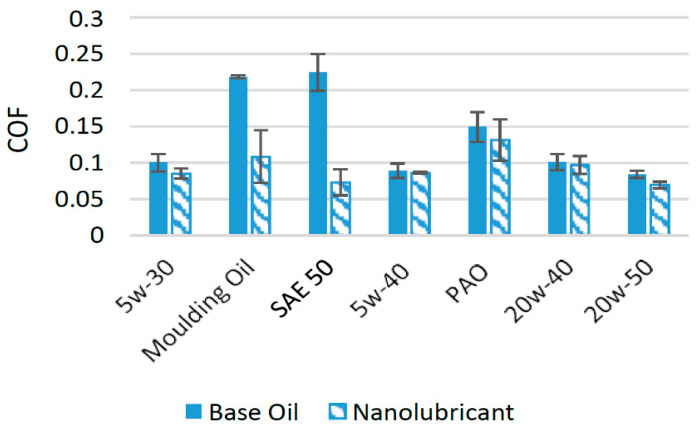
Effect of MoS_2_ NPs on the friction performance of synthetic lubricants.

In the past few years, molybdenum disulfide has appeared as a potential source for reducing the wear and friction associated with different lubricants [54]. A large number of experimental investigations have been performed to assess the effect of MoS_2_ nanoparticles on the wear and friction performance of different lubricants. In one such study, nanoparticles of MoS_2_ were used as an additive in SAE 5w-30 engine oil at different concentrations. The results of the tribological tests using a four-ball configuration showed that when 0.5 wt% of MoS_2_ was used as an additive, the COF was minimal. When compared to oil with no nanoparticle additive, the COF was reduced by almost 15% at this optimum concentration [51]. MoS_2_ nanoparticles positively affected the friction performance of molding oil when used in a piston skirt-liner tribometer. The COF was greatly reduced by using MoS_2_ nanoparticles as an additive. The minimum COF was found when 1 wt% of MoS_2_ nanoparticles were used as an additive. At this optimum concentration, the COF decreased by almost 50% in comparison to molding oil with no nanoparticle additive [22]. In another study, HD 50 Engine Oil (SAE 50) was used as a lubricant in the presence of MoS_2_ nanoparticles on a pin-on-disk tribotester. The addition of MoS_2_ nanoparticles enhanced the tribological properties of the lubricant and helped to reduce the COF and wear of the interacting surfaces. The concentration of nanoparticles in the lubricant ranged between 0.25 and 1.25 wt% and the optimum concentration was found to be 1 wt% of MoS_2_ in HD 50 engine oil. The nanoparticles tended to agglomerate at concentrations higher than the optimum concentration. The COF was found to be reduced by 67.6% compared to oil with no nanoparticle additive [23]. MoS_2_ nanoparticles were not able to enhance the friction performance of the SAE 5w-40 engine oil as much under the tested conditions on a four-ball tribotester. Two concentrations, 0.15 wt% and 0.20 wt%, of MoS_2_ nanoparticles were tested as additives in SAE 5w-30 engine oil. Among these concentrations, 0.20 wt% of MoS_2_ appeared as the optimum concentration and reduced the COF by almost 3.37% [55]. In another experimental study, MoS_2_ nanoparticles were used at a concentration of 0.5 wt% in polyalphaolefin (PAO) on a four-ball tribotester. The results of the experiment showed that the COF was reduced by 12.24% compared to PAO with no nanoparticle additive [56]. MoS_2_ nanoparticles were employed as an additive in SAE 20w-40 oil at four different particle concentrations ranging between 0.25 and 1 wt%, and the optimum concentration was found to be 0.50 wt% of MoS_2_. The COF was reduced by 16.44% at this optimum concentration, while at higher concentrations, the reduction in COF was not as evident due to the increased agglomeration of the nanoparticles [57]. The addition of MoS_2_ nanoparticles reduced the COF of interacting surfaces (pin-on-disk) by almost 17.11% when used at a concentration of 0.50 wt% in SAE 20w-50 [58]. Figure 8 shows the comparison of results when MoS_2_ nanoparticles were used as an additive in different lubricants.

The comparison in Figure 8 shows that MoS_2_ nanoparticles perform much better with SAE 50 and greatly reduce the average COF, which shows that MoS_2_ nanoparticles are highly compatible with SAE 50. The capability of MoS_2_ nanoparticles to reduce the COF in the tested lubricants can be accredited to the conversion of sliding friction to rolling friction, where MoS_2_ produces a three-body rolling effect [59]. Moreover, they also help in the formation of tribofilms between interacting surfaces, which ultimately result in a reduction in the COF and wear compared to oils with no nanoparticle additive [60,61].

### 3.7. Multiwalled Carbon Nanotubes (MWCNTs)

**Figure 9:**
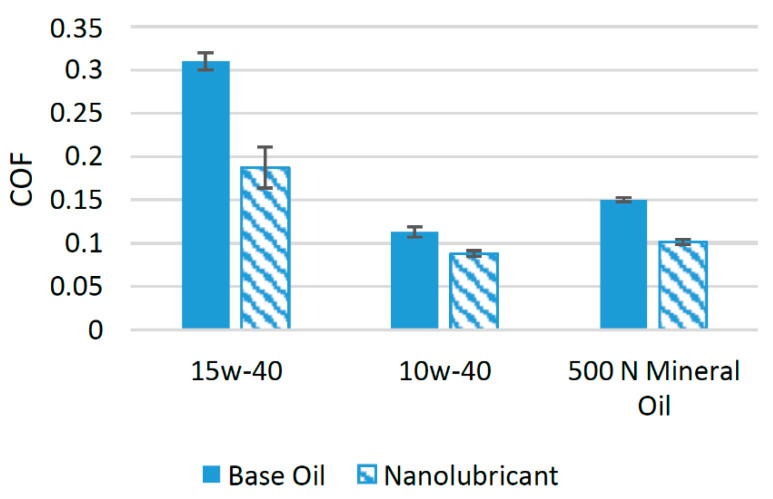
Effect of MWCNTs on the friction performance of synthetic lubricants.

MWCNTs have emerged as a potential source to reduce the wear and friction between interacting surfaces [62]. In the past few years, a number of researchers related to the field of tribology have focused their intentions on finding the tribological effect of adding MWCNTs to different lubricants. In a previous study, researchers used MWCNTs as an additive in SAE 15w-40. Tribological tests using a pin-on-disk configuration were carried out by adding five different MWCNT concentrations ranging between 0.3 and 1.3 wt%. The results of the experiment showed that when 1 wt% of MWCNTs was used as an additive, the COF was reduced to a minimum. At this optimum concentration, the COF was reduced by 39.55% when compared to oil with no nanoparticle additive [16]. In another study, MWCNTs were added to SAE 10w-40 oil to evaluate their effects on the tribological performance of the mentioned oil using a four-ball tribotester. The concentration at which MWCNTs were used was reported to 0.18 wt%. When compared to the oil with no nanoparticle additive, the COF was reduced by almost 22.12% at a concentration of 0.18 wt% [63]. The effects of the addition of MWCNTs in 500 N mineral oil were investigated in an experimental study, in which 0.20 wt% of MWCNTs were added to the mentioned oil. The results of the test showed that the COF was reduced by 32.47% when compared to the oil with no nanoparticle additive [64]. Figure 9 shows a comparison of the average COF when MWCNTs were used as an additive in different lubricants. It can be seen that MWCNTs reduced the COF by a high percentage when used with SAE 15w-40 compared to other lubricants. The reduction in the COF of the interacting surfaces resulting from adding MWCNTs to lubricant oil was attributed to their high young modulus, which enables them to shear as a result of a high friction force. MWCNTs have the potential to be adsorbed on rubbing surfaces, which helps to avoid metal-to-metal to contact. Moreover, the sliding or rolling associated with MWCNTs between the interacting surfaces results in a reduction in the COF and enhances the wear resistance [65].

### 3.8. Copper Oxide (CuO) Nanoparticles

**Figure 10:**
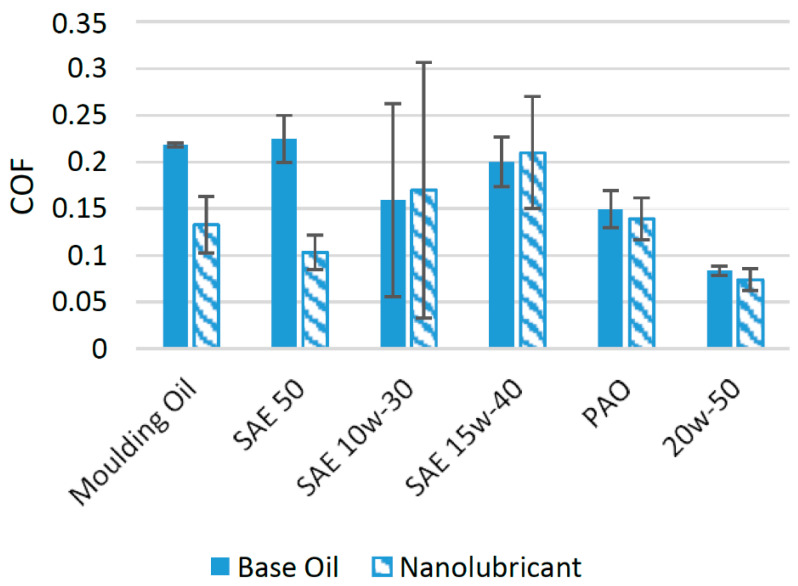
Effect of CuO NPs on the friction performance of synthetic lubricants.

Among the nanoparticles used as friction modifiers, CuO nanoparticles are the most abundantly used. Extensive experimental studies have been performed to examine the effects of these nanoparticles on the wear, friction, and overall tribological characteristics of different lubricants. In an experimental study, CuO nanoparticles were added at different concentrations ranging between 1 and 2 wt% to molding oil. The results of the tribological test with a pin-on-disk configuration showed that when 1.3 wt% of CuO nanoparticles were employed as an additive, the COF reduced to a minimum and started increasing when the nanoparticle concentration increased further. This increase in the COF after attaining a minimum value at a specific concentration was associated with the agglomeration of the nanoparticles due to their increased concentration. On average, the COF was reduced by almost 39.22% compared to molding oil with no nanoparticle additive [66]. CuO nanoparticles exhibited good friction performance when used with HD 50 Engine Oil (SAE 50), when a concentration ranging between 0.25 and 1.45 wt% was employed as an additive. The results of the tribological test using a pin-on-disc tribotester showed that the COF was reduced to a minimum when 1 wt% of CuO nanoparticles was used, while the agglomeration of nanoparticles at concentrations above 1 wt% resulted in an increased COF. At the optimum concentration of nanoparticles, the COF was reduced by 53.98% compared to engine oil with no nanoparticle additive [23]. Nanoparticles of CuO were not able to modify the friction performance of SAE 10w-30 oil in a positive manner. The resulting average COF when CuO nanoparticles were used as an additive was increased compared to oil with no nanoparticle additive under tested experimental conditions. Through statistical analysis, it was found that when 0.0086 wt% of CuO nanoparticles was used as an additive in SAE 10w-30 and a 75.152 N load was applied at a rotational speed of 291.3360 rpm, the COF could be reduced to a minimum [67]. On average, the COF was increased when CuO nanoparticles were deployed as additives in SAE 15w-40 using a four-ball tribotester. The COF was found to be decreased compared to oil with no nanoparticle additive when 0.1 wt% of CuO nanoparticles was used and the temperature of the experimentation was kept at 60 °C. However, on other tested conditions, SAE 15w-40 resulted in a lower COF compared to oil with an additive [68]. PAO showed synergetic relation with CuO nanoparticles, resulting in a reduced COF compared to PAO with no nanoparticle additive. It was found that when 0.5 wt% of CuO nanoparticles was used in PAO, the COF was reduced by 6.96% compared to PAO with no nanoparticle additive [52]. The COF resulting by using CuO nanoparticles in SAE 20w-50 was found to be reduced compared to oil with no nanoparticle additive. Tribological experiments using a pin-on-disk configuration were conducted using nanoparticles at three different concentrations ranging between 0.25 and 1 wt%. The results showed that the COF was reduced by 11.14% compared to oil with no nanoparticles when 0.25 wt% of CuO nanoparticles was used [58]. Figure 10 shows an average effect of CuO nanoparticles on the COF when used in different lubricant oils. The reduction in the average COF resulting from the use of CuO nanoparticles could be associated with the development of a three-body layer effect between the interacting surfaces [69]. Moreover, the size and hardness associated with CuO nanoparticles also participated in reducing the friction and wear between the interacting surfaces [70]. CuO nanoparticles showed extremely good performance with molding oil and HD 50 Engine oil, while these nanoparticles were not able to perform well with SAE 10w-30 and SAE 15w-40 under certain operating conditions. Thus, there is a need to perform further tribological tests with a diverse range of operating conditions to properly predict this negative behavior of CuO nanoparticles on both lubricant oils.

### 3.9. Alumina (Al_2_O_3_) Nanoparticles

**Figure 11:**
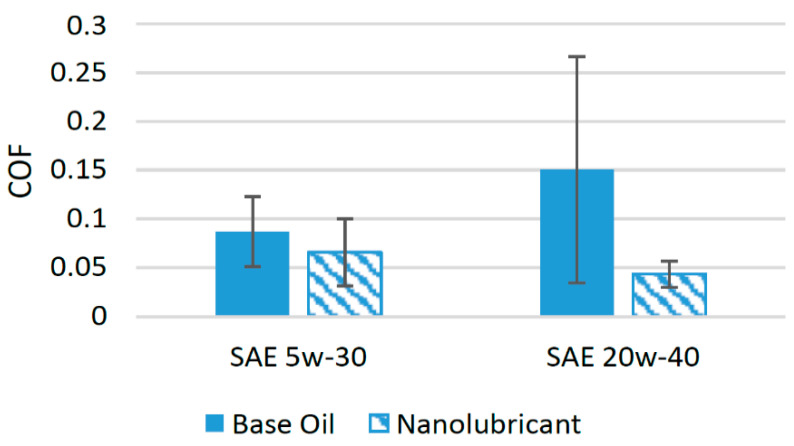
Effect of Al_2_O_3_ NPs on the friction performance of synthetic lubricants.

A few studies have been performed in order to evaluate the tribological performance of alumina nanoparticles with different lubricants in recent years [71]. The effects of alumina on the tribological performance of SAE 5w-30 lubricant oil was investigated in a previous study. These nanoparticles improved the friction performance, and the COF was reduced by almost 24.22% in comparison to oil with no nanoparticle additive in a tribological test conducted using piston ring assembly [72]. In another study, alumina nanoparticles were used with SAE 20w-40 oil in a pin-on-disk tribometer to investigate its impact on the tribological performance. A set of eighteen tests was conducted and the concentration, applied load, rotation speed, and size of the nanoparticles were varied. The results of these tribological tests showed that when nanoparticles with a size of 60 nm were used at a concentration of 1 wt% under a load of 160 N and an operating speed of 800 rpm, the COF was reduced to a minimum. Under these operating conditions, the COF was found to be 0.0256, almost 59.17% less than oil with no nanoparticle additive [73]. This decline in COF due to the presence of alumina nanoparticles between the interacting surfaces was because of the inherent ball-bearing effect, the mending effect, and the formation of a thin tribofilm. The tribofilm developed as a result of a chemical interaction between alumina nanoparticles, lubricant oil, and the wear debris [73]. Figure 11 shows the effect of the addition of alumina nanoparticles on friction performance of different lubricants.

It can be seen that the alumina nanoparticles performed efficiently with SAE 20w-40 and reduced the COF by a higher percentage compared to SAE 5w-30, which shows that alumina is highly suitable for use as a friction modifier in SAE 20w-40.

### 3.10. Silica (SiO_2_) Nanoparticles

**Figure 12:**
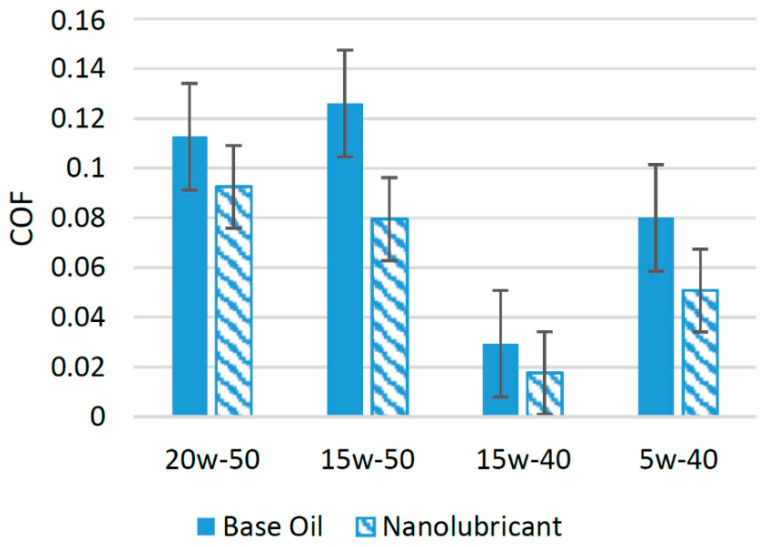
Effect of SiO_2_ NPs on the friction performance of synthetic lubricants.

Silica nanoparticles have emerged as good source to modify the tribological properties of different lubricants. A number of studies have been conducted in recent years in order to validate the synergetic behavior of silica nanoparticles with different lubricant oils. In an experimental study, silica nanoparticles were used with SAE 20w-50 oil. The experiments were conducted using samples with two different concentrations of nanoparticles. One of the samples contained 0.5 wt% of SiO_2_, while the other contained 1 wt% of SiO_2_. The outputs of the tribological tests were compared with outputs from oil with no nanoparticle additive. The results showed that when 0.50 wt% of silica nanoparticles was used, the COF was reduced to a minimum. On average, by using silica nanoparticles as an additive, the COF was reduced by almost 17.89% compared to oil with no nanoparticle additive. In the same study, SAE 15w-50 was also used to check the suitability of adding silica nanoparticles. The same two concentrations were used in this case as well. The results of the tribological tests with a pin-on-disk configuration indicated that when 1 wt% of silica nanoparticles was used, the COF was reduced to a minimum compared to oil with no nanoparticle additive. On average, the silica nanoparticles were able to reduce the COF associated with SAE 15w-50 by almost 36.84% [21]. Four different samples were prepared by adding 0.25, 0.5, 0.7, and 1 wt% of silica nanoparticles in SAE 5w-40. The results of the tribological tests using a ball-on-plate configuration showed that when 0.7 wt% of silica nanoparticles was used, the COF was reduced to a minimum compared to oil with no nanoparticle additive. On average, the silica nanoparticles were able to reduce the resulting COF by almost 36.56% [9]. Figure 12 shows the variation in the COF related to different lubricants when silica nanoparticles were used as an additive. SiO_2_ nanoparticles filled the valleys between the interacting surfaces and resulted in a lower COF. The deep grooves and valleys on the interacting surfaces were filled by silica nanoparticles, which adhered there and reduced COF, forming a protective layer [74]. SAE 15w-50 performed extremely well with silica nanoparticles, and the COF was reduced by a higher percentage in this case compared to other lubricants. This shows that silica nanoparticles are highly compatible with SAE 15w-50 oil.

### 3.11. Tungsten Disulfide (WS_2_) Nanoparticles

**Figure 13:**
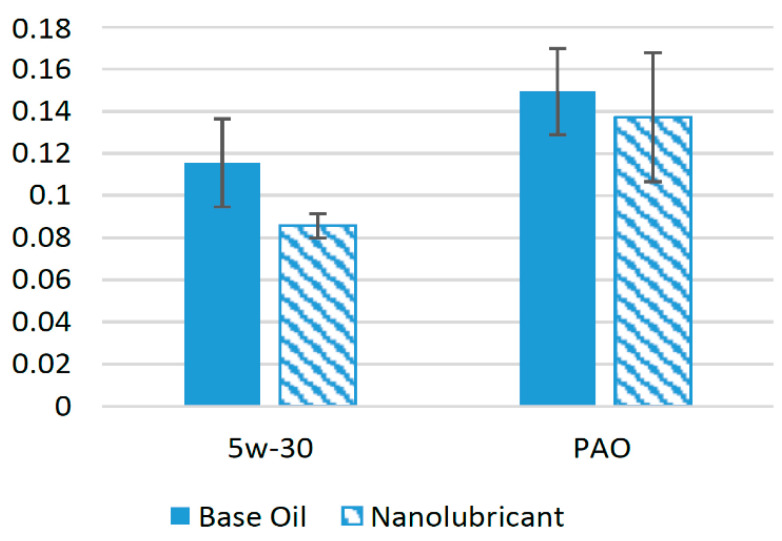
Effect of WS_2_ NPs on the friction performance of synthetic lubricants.

A few studies have been conducted on the usage of WS_2_ nanoparticles with several lubricants. In one such study, WS_2_ nanoparticles were used with SAE 5w-30 oil on a four-ball tribotester in order to determine its tribological behavior with the mentioned oil. The authors also investigated the influence of particle size on the overall tribological properties. Samples with different concentrations of micro- to nano-sized particles of WS_2_ were prepared. The results showed that nanoparticles were better than micro-particles at reducing the friction coefficient. On average, the COF was found to be reduced by almost 25.89% when WS_2_ nanoparticles were used compared to oil with no nanoparticle additive, while the minimum COF was found at 1 wt% of WS_2_ nanoparticles [75]. In another experimental study, the tribological behavior of WS_2_ nanoparticles was investigated with PAO using a four-ball tribotester. A sample containing 0.5 wt% of WS_2_ nanoparticles was used in a tribological test, and its effect on the COF and wear was investigated. The results of the test showed that, on average, the COF was reduced by almost 8.17% compared to oil with no nanoparticle additive [56]. Figure 13 shows the effect on the COF associated with different lubricants when WS_2_ nanoparticles were used as an additive. The boundary friction was reduced due to the laminar structure of the tribofilm associated with WS_2_. Moreover, WS_2_ nanoparticles filled the gaps and covered the reacted tribofilm, making it a potential source for COF reduction [76].

## 4. Nanoparticles in Biolubricants

Biolubricants have attracted significant attention in the past few years due to their sustainability and environmentally friendly properties. It has been found that biolubricants have the potential to replace conventional lubricants [77]. A number of nanoparticles have been investigated with biolubricants in the past five years, but these studies are much lower in number compared to studies conducted with synthetic oils. Among the nanoparticles discussed above, CuO and MoS_2_ are most the widely used in biolubricants. CuO nanoparticles show extremely good performance with biolubricants and reduce the COF of the interacting surfaces by high percentages compared to biolubricants with no nanoparticle additive. Although a large number of nanoparticles have been used as friction modifiers in biolubricants, there are many nanomaterials that still need to be tested with biolubricants. Graphene nanoparticles have shown positive behavior and an increase in friction resistance when used with synthetic lubricants. However, the effect of graphene nanoparticles on the friction performance of biolubricants is not well established in the literature. Thus, there is a need to test such nanoparticles with biolubricants to fully understand their effect on the friction performance of biolubricants. It has also been found in some studies that when hybrid nanoparticles (combination of two or more nanomaterials) are used as an additive, the COF is reduced by a higher percentage compared to individual nanomaterials. Therefore, there is a need to conduct experimental studies to examine the effect of such hybrid nanoparticles on the tribological performance of biolubricants. Moreover, actual engines need to be tested with biolubricants with nanoparticles as additives to check the suitability of such lubricants under actual operating conditions. The effect on the tribological properties of biolubricants by the addition of nanoparticles is discussed in this section.

### 4.1. Titanium Dioxide (TiO_2_) Nanoparticles

**Figure 14:**
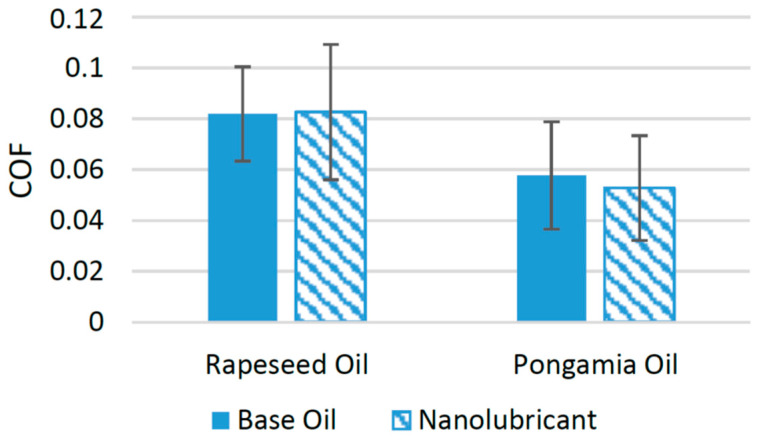
Effect of TiO_2_ NPs on the friction performance of bio-lubricants.

Figure 14 shows the variation in COF when TiO_2_ nanoparticles were used in different base oils. It has been found that TiO_2_ nanoparticles are unable to perform positively with rapeseed oil. For example, in a previous study, the COF was increased under the tested range of operating conditions using a four-ball tribotester compared to neat rapeseed oil, and it was suggested that experiments must be conducted with some other sets of operating conditions in order to find the suitability of TiO_2_ nanoparticles with rapeseed oil [78]. Meanwhile, the addition of TiO_2_ nanoparticles in pongamia oil reduced the COF by almost 10% when an optimum concentration of 0.1 wt% of nanoparticles was used on a pin-on-disk tribometer. This decrease in the coefficient of friction was accredited to the deposition of nanoparticles on the interacting surfaces, which resulted in a strong protective film between the interacting surfaces [79]. Although these nanoparticles influenced the tribological properties of biolubricants, their performance with synthetic lubricants was much better with synthetic oils, as discussed previously.

### 4.2. Zinc Oxide (ZnO) Nanoparticles

**Figure 15:**
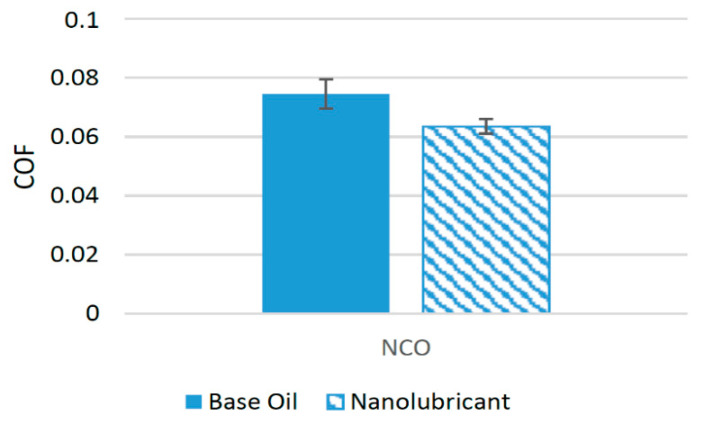
Effect of ZnO NPs on the friction performance of bio-lubricants.

The literature shows that ZnO nanoparticles can be employed to enhance the tribological properties of biolubricants in only few a studies. These nanoparticles perform well when used with natural castor oil (NCO) and reduce the COF, resulting from rubbing surfaces. Experiments have been performed under different operating conditions, with the concentration of nanoparticles in lubricant samples varying. For example, tribological tests were conducted at four different concentrations of nanoparticles using a four-ball test rig. The concentrations used were 0.1 wt%, 0.5 wt%, 1 wt%, and 2 wt% of ZnO. The results of the test showed that when 0.1 wt% of ZnO nanoparticles was used as an additive, the COF was reduced to a minimum, while on average, ZnO nanoparticles reduced the COF by 14.74% compared to NCO with no nanoparticle additive [6]. Figure 15 shows the effect of the addition of nanoparticles on the average COF associated with NCO. This synergetic behavior of ZnO nanoparticles can be associated with ZnO nanoparticle’s ability to form a tribofilm between the interacting surfaces and to produce a three-body rolling effect that turns sliding friction into rolling friction [80]. Only a few experimental studies have been performed to evaluate the friction performance of ZnO nanoparticles with biolubricants. Thus, there is a need to conduct experimental studies using ZnO nanoparticles as a friction modifier with different biolubricants.

### 4.3. Hexa-Boron Nitride (h-BN) Nanoparticles

**Figure 16:**
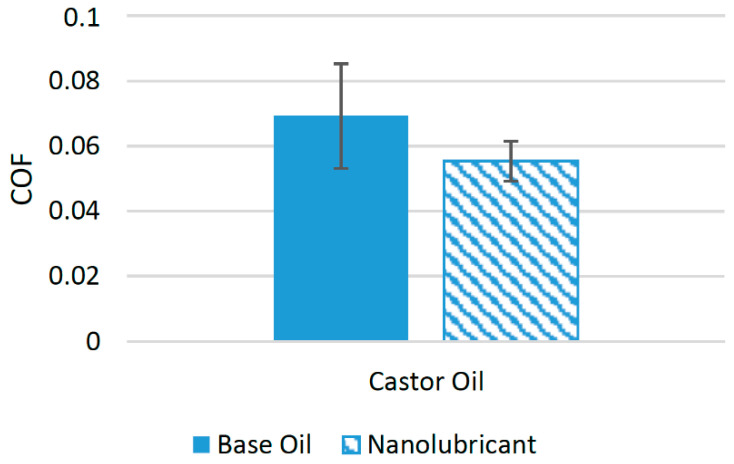
Effect of h-BN NPs on the friction performance of bio-lubricants.

Like the nanoparticles previously discussed, h-BN nanoparticles are not used widely in biolubricants in the literature. Only a few experimental studies have been conducted in which h-BN nanoparticles were used as an additive to reduce the COF of the interacting surfaces. In one of the experimental studies, h-BN nanoparticles were used with castor oil to examine the effects of the addition of h-BN nanoparticles. Four different concentrations of h-BN nanoparticles were used as an additive, namely, 1 wt%, 2 wt%, 5 wt%, and 8 wt%, and tribological tests using a ball-on-plate configuration were performed to find the effect of these concentrations on the performance of the lubricant. The optimum concentration of h-BN nanoparticles for castor oil was found to be 1 wt%. It was found that the COF was reduced by almost 30.2% at this optimum concentration, while at a higher concentration, the reduction in the COF was not as high because the nanoparticles agglomerated at higher concentrations [81]. Figure 16 shows a comparison of the average COF with and without nanoparticles in castor oil. The positive behavior of these nanoparticles with castor oil can be associated to the potential of these nanoparticles to establish a tribochemical film on the interacting surface. It has been found that these nanoparticles can form a boron oxide (B_2_O_3_)-based tribofilm, which helps to reduce the COF of interacting surfaces [18]. Although a few experimental studies have been performed to check the suitability of h-BN nanoparticles as a friction modifier, there are still a number of biolubricants that are yet to be considered in order to fully predict the suitability of these nanoparticles as friction modifiers for biolubricants.

### 4.4. Molybdenum Disulfide (MoS_2_) Nanoparticles

**Figure 17:**
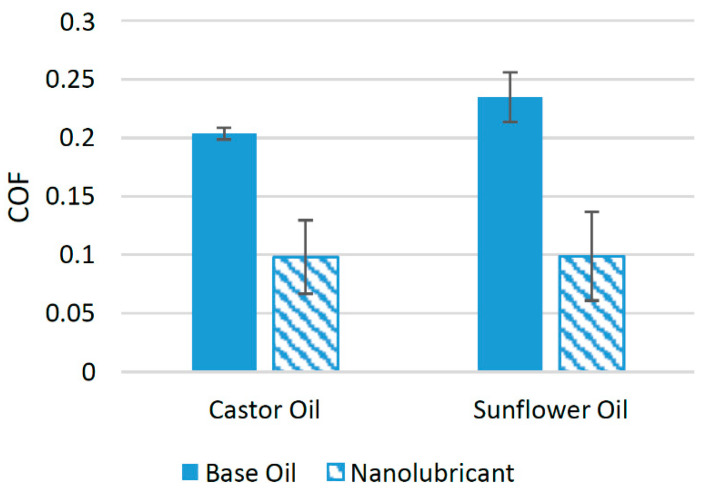
Effect of MoS_2_ NPs on the friction performance of bio-lubricants.

MoS_2_ nanoparticles have been used as friction modifiers in a few biolubricants [82]. In one such study, MoS_2_ nanoparticles were used in castor oil to check their suitability as friction modifiers. Two samples containing 1 wt% and 2 wt% of MoS_2_ were used. Tribological tests using a pin-on-disk configuration showed that when 1 wt% of MoS_2_ nanoparticles was used as an additive, the COF was reduced to a minimum. At this optimum concentration, the COF was reduced by almost 81.7%, while, on average, the COF was found to be reduced by 51.76% compared to castor oil with no nanoparticle additive [66]. In another study, MoS_2_ nanoparticles were used as a potential source to reduce the COF associated with sunflower oil. MoS_2_ nanoparticles performed tremendously well with sunflower oil and reduced the COF by a huge percentage. Five samples were prepared with concentrations of nanoparticles ranging between 0.1 and 1.25 wt%. The results of the tribological tests using a pin-on-disk tribotester showed that the COF was reduced to a minimum when 1 wt% of MoS_2_ nanoparticles was used, and started increasing at higher concentrations due to the increased agglomeration of nanoparticles at such concentrations. On average, the COF was reduced by almost 57.89% compared to oil with no nanoparticle additive [23]. Figure 17 shows the effect of MoS_2_ nanoparticles on the COF of castor oil and sunflower oil. It can be seen that in both cases, the COF was greatly reduced compared to oils with no nanoparticle additive, which shows that the subjected nanoparticles are compatible with both base lubricants. The positive behavior of MoS_2_ nanoparticles in terms of reducing the COF of biolubricants can be attributed to their capability of forming molybdenum (Mo)- and sulfur (S)-based tribofilms between interacting surfaces, which helps to reduce the COF of interacting surfaces [83]. Moreover, MoS_2_ nanoparticles also tend to fill the valleys formed by the asperities on the surface, which also helps in the reduction of the COF [84]. Although MoS_2_ nanoparticles have shown good performance with the discussed biolubricants, there are still a number of biolubricants that are yet to be tested with MoS_2_ nanoparticles to declare them as a friction modifier for the biolubricants.

### 4.5. Copper Oxide (CuO) Nanoparticles

**Figure 18:**
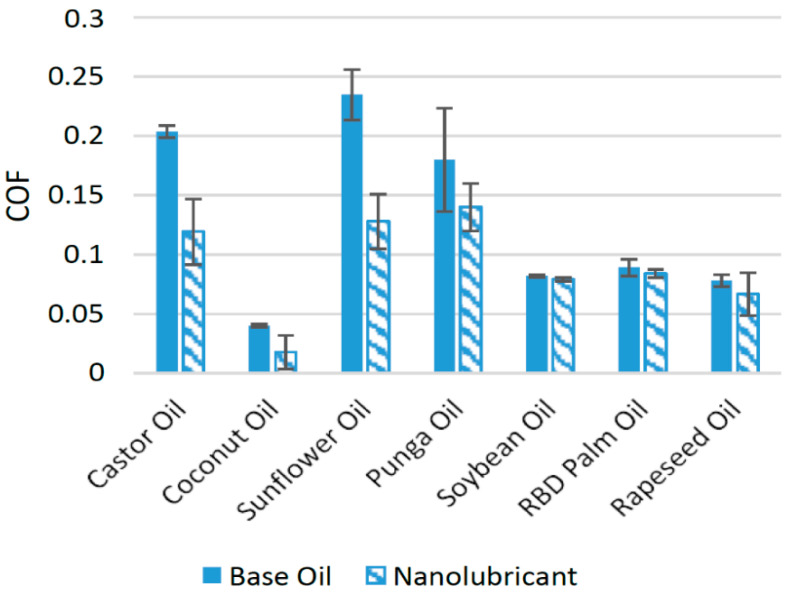
Effect of CuO NPs on the friction performance of bio-lubricants.

Amongst the nanoparticles used in biolubricants, CuO has been the most common in the last five years. Nanoparticles of CuO perform well with most biolubricants considered [85,86]. In an experimental study, CuO nanoparticles were used with castor oil. Two samples were prepared, one with 1 wt% and the other 2 wt% of CuO nanoparticles. Tribological experiments using a pin-on-disk arrangement were conducted, subjected to various operating conditions, and the results of the tests were compared to those of oil with no nanoparticle additive. The results of the test showed that the COF was reduced to a minimum when 1 wt% of CuO nanoparticles was used as an additive, while the COF increased at higher concentrations. At an optimum concentration, the COF was found to be reduced by almost 76.02% compared to castor oil without nanoparticles, while, on average, the COF was reduced by 41.42% compared to base castor oil [66]. Coconut oil with no nanoparticle additive showed a very low COF compared to other biolubricants. Nanoparticles of CuO reduced the COF of coconut oil even further. Five samples with concentrations of nanoparticle ranging between 0.25 and 1.25 wt% were used as an additive. The results of the tribological tests using a block-on-plate test configuration showed that the COF was reduced to the minimum when 0.5 wt% of CuO nanoparticles was used. On average, CuO nanoparticles were able to reduce the COF of coconut oil by almost 55.55% compared to coconut oil without nanoparticles [87]. In another experimental study, the tribological suitability of CuO nanoparticles was checked with sunflower oil. Five samples with nanoparticle concentrations ranging between 0.25 and 1.25 wt% were used. The COF was found to be reduced to a minimum when 1 wt% of CuO nanoparticles was used as an additive. On average, the COF was found to be reduced by almost 45.49% compared to sunflower oil with no nanoparticle additive when used in a pin-on-disc tribotester [23]. Punga oil also performed well with CuO nanoparticles. The COF was greatly reduced when CuO nanoparticles were employed as an additive in punga oil. In an experimental study, CuO nanoparticles were used in different concentrations in punga oil to check the suitability of CuO nanoparticles as a friction modifier using a pin-on-flat arrangement of the tribometer. The COF was found to be minimum when 1 wt% of CuO nanoparticles was employed as an additive. On average, the nanolubricant was able to reduce the COF of the interacting surfaces by 22.22% compared to punga oil with no nanoparticle additive [68]. In another study, soybean oil was used as a base oil and its compatibility with CuO nanoparticles was evaluated using a four-ball tribotester. The COF of the nanolubricant was reduced compared to soybean oil with no nanoparticle additive, but this reduction in the COF was not so prominent. On average, the COF of the nanolubricant was reduced by 3.66% compared to soybean oil with no nanoparticle additive [88]. In the same experimental study, RBD (refined, bleached, and deodorized) palm oil was also used as a test base lubricant. Similarly to the case of soybean oil, the COF reduction was not as prominent in this case either. Still, on average, the COF in the case of the nanolubricant was reduced by almost 5.62% compared to RBD palm oil with no nanoparticle additive [88]. The friction performance of rapeseed oil was improved by using CuO nanoparticles as an additive. Tribological tests were conducted using five samples of nanoparticles with concentrations ranging between 0.1 and 1 wt%. The results from a four-ball tribotester showed that the COF was reduced to a minimum when 0.50 wt% of CuO nanoparticles was used as an additive in rapeseed oil. On average, CuO nanoparticles were able to reduce the COF by almost 14.67% compared to rapeseed oil without a nanoparticle additive [20]. Figure 18 shows a comparison of the COF when CuO nanoparticles were employed as additives in different biolubricants. It can be seen that the nanolubricant performed well and reduced the COF in each case, and showed the best performance with sunflower oil. From the comparison shown in Figure 18, it can be established that CuO nanoparticles can be employed as a potential source to reduce the COF. This positive behavior associated with CuO nanoparticles can be attributed to the ball-bearing effect produced by CuO nanoparticles [89]. Moreover, CuO nanoparticles also have the potential to adhere to the interacting surfaces and filling the valleys formed by large asperities, which also helps in reducing the COF [90]. It can be seen that CuO nanoparticles reduced the average COF by higher percentages when used with castor oil and sunflower oil, which shows that CuO nanoparticles are highly compatible with lubricants compared to others.

### 4.6. Alumina (Al_2_O_3_) Nanoparticles

**Figure 19:**
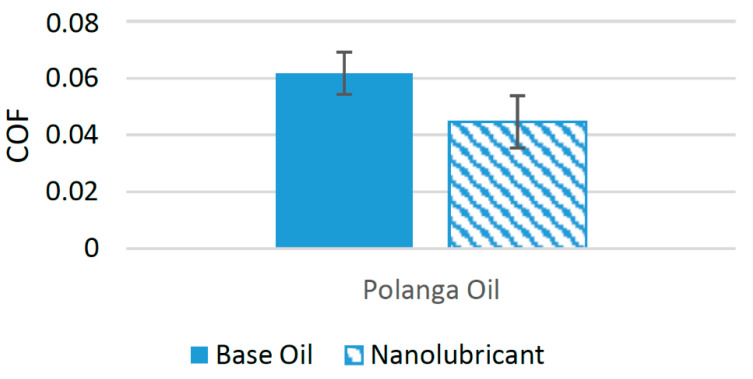
Effect of Alumina (Al_2_O_3_) NPs on the friction performance of bio-lubricants.

Alumina nanoparticles have been used in a few studies to enhance the tribological performance of biolubricants. In one of these studies, alumina nanoparticles were used as a friction modifier in polanga oil. Four different samples were prepared. The concentration of nanoparticles in the base oil ranged between 0.01 and 0.1 wt%. Tribological tests employing a four-ball configuration were conducted using these samples, and the results were compared with those of polanga oil with no nanoparticle additive. The results of the tests showed that the COF was reduced to a minimum when 0.08 wt% of alumina nanoparticles was used. On average, alumina nanoparticles were able to reduce the COF of the interacting surfaces by almost 27.76% compared to polanga oil with no nanoparticle additive [91]. Figure 19 shows the effect of the addition of nanoparticles on the COF. The positive behavior associated with the use of alumina nanoparticles can be associated with the accumulation of nanoparticles in the valleys of the interacting surfaces and the formation of a tribofilm at the interacting surface [92]. There are not enough studies in the literature where alumina nanoparticles have been used as friction modifiers in biolubricants. Only a few experimental studies have been conducted in the last five years. Thus, there is a need to carry out further experimental studies using alumina nanoparticles in different biolubricants to properly compare their effects on friction performance when used with different biolubricants.

### 4.7. Silica (SiO_2_) Nanoparticles

**Figure 20:**
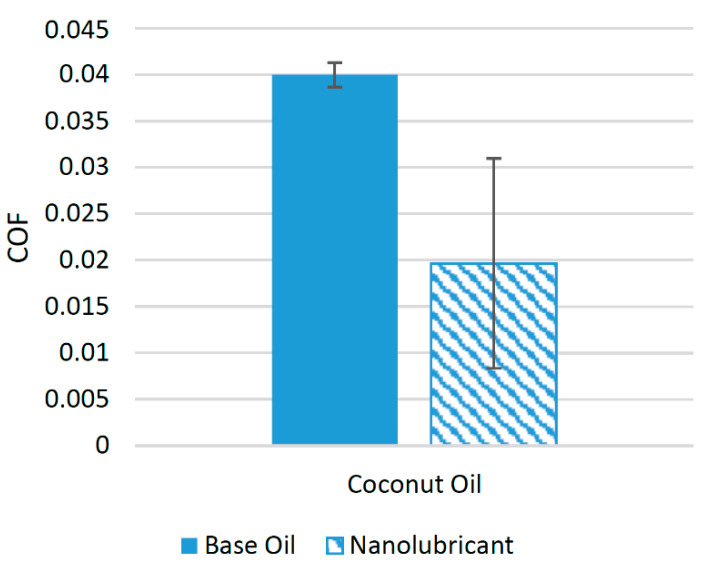
Effect of Silica (SiO_2_) NPs on the friction performance of bio-lubricants.

A limited number of studies have been conducted in recent years to check the suitability of silica nanoparticles with biolubricants. In an experimental study, silica nanoparticles were used with coconut oil, using a block-on-ring configuration. Five samples were prepared with different concentrations of nanoparticles. The concentrations ranged between 0.25 and 1.25 wt% of silica nanoparticles. The results of the experiment showed that the COF was reduced to a minimum when 1 wt% of silica nanoparticles was used. On average, silica nanoparticles were able to reduce the COF of the interacting surface by almost 50.85% compared to coconut oil with no nanoparticle additive [90]. Figure 20 shows the effect of the addition of nanoparticles on the COF. Although silica nanoparticles performed well with coconut oil, there is still a need to check the suitability of silica nanoparticles for other biolubricants as well. The positive behavior associated with the use of silica nanoparticles on the COF could be a result of the deposition of silica nanoparticles on the interacting surfaces [93].

Among the nanoparticles discussed above, CuO and MoS_2_ are most widely used in biolubricants. CuO nanoparticles show extremely good performance with biolubricants and reduce the COF of interacting surfaces by high percentages compared to biolubricants with no nanoparticle additive. Although a large number of nanoparticles have been used as friction modifiers in biolubricants, there are many nanomaterials that still need to be tested with biolubricants. Graphene nanoparticles show positive behavior and increase the friction resistance when used with synthetic lubricants. However, the effect of graphene nanoparticles on the friction performance of biolubricants is not well established in the literature. Thus, there is a need to test such nanoparticles with biolubricants to fully understand their effect on the friction performance of said biolubricants. It has also been found in some studies that when hybrid nanoparticles (combination of two or more nanomaterials) are used as an additive, the COF is reduced by a higher percentage compared to individual nanomaterials [94]. Therefore, there is a need to conduct experimental studies to examine the effect of such hybrid nanoparticles on the tribological performance of biolubricants. Moreover, actual engines need to be tested with biolubricants with nanoparticles as additives to check the suitability of such lubricants under actual operating conditions.

## 5. Limitations of Nanolubricants

Although nanoparticles have the potential to enhance the tribological properties of lubricants and to reduce the COF and wear of interacting surfaces, there are certain limitations in terms of using nanoparticles as additives. One such limitation is associated with the use of nanoparticles is agglomeration, which deteriorates their positive effect on the COF and wear. Agglomeration refers to the grouping of nanoparticles to form larger particles with less surface energy and a high surface area [95]. It has been found in many studies that when the concentration of nanoparticles is increased beyond the optimum concentration, agglomeration of the nanoparticles starts and the tribological performance of lubricants starts deteriorating [96,97]. Another problem associated with use of nanoparticles is their dispersion stability in lubricant oil, which adversely affects the tribological performance of this oil [75]. The dispersion stability of nanoparticles in lubricant oils can be improved by using suitable surfactants and surface modification techniques [98,99].

## 6. Conclusions

This paper showcased a comprehensive review on the use of nanoparticles as lubricant additives by comparing their effect on the friction performance of different synthetic and biolubricants. The COF (coefficient of friction), mechanisms responsible for improvements in tribological properties, and limitations with the use of nanoparticles were thoroughly discussed. It was found that among the nanoparticles considered, CuO (copper oxide), molybdenum disulfide (MoS_2_), and titanium dioxide (TiO_2_) show better tribological performance with lubricant oils compared to other nanoparticles. From a comparison, it was found that the most compatible lubricants for MoS_2_ are SAE 50 oil and sunflower oil. CuO nanoparticles also showed higher compatibility with both SAE 50 oil and sunflower oil compared to other lubricants. TiO_2_ nanoparticles were found to be the most suitable in order to reduce friction when used with SAE 10w-30 oil and pongamia oil. However, there is a need to conduct more research regarding the use of nanoparticles as additives in biolubricants. Moreover, the effects of nanolubricants on the performance of actual engines need to be studied.
